# Stigmatized Stroke? A Qualitative Study of Perception of Stroke Among Community Residents With Hypertension

**DOI:** 10.3389/ijph.2024.1606781

**Published:** 2024-03-13

**Authors:** Meijuan Wan, Xiaoxu Liu, Mengdi Zhang, Zixin Cui, Wenjuan Zhao, Jianzhou Li, Shumei Lin

**Affiliations:** ^1^ Department of Infectious Diseases, The First Affiliated Hospital of Xi’an Jiaotong University, Xi’an, Shaanxi, China; ^2^ Department of Physiology and Pathophysiology, School of Basic Medicine, Xi’an Jiaotong University, Xi’an, Shaanxi, China

**Keywords:** stigma, stroke, hypertension, perception, public attitudes

## Abstract

**Objectives:** To understand the perception of stroke in the hypertensive population. Hypertension is the primary risk factor for stroke, and current approaches to stroke prevention are inadequate and often fragmented. Understanding the perception of stroke among individuals with hypertension is crucial for a targeted approach. However, empirical evidence on this perception is limited.

**Methods:** A qualitative design involved thematic analysis of focus groups and interview data from urban China with hypertension. Audio recordings were transcribed and subjected to thematic analysis.

**Results:** Three themes were identified. Hypertensive participants first *identified stroke* patients by their *obvious physical disability*, and then identified the disease as *a negative thing*. Finally, they wanted to *stay away from stroke*, but paradoxically, there is a contradictory approach to *avoidance* and *prevention,* such as being willing to prevent the disease or simply avoiding socializing with stroke patients.

**Conclusion:** Hypertensive patients hold complex and diverse perceptions of stroke, including a certain stigma. Future public health education should prioritize improving media promotion and fostering interaction between patients with hypertension and stroke in the community.

## Introduction

Stroke is the second-most common cause of death and one of the leading contributors to long-term disability worldwide [1]. Although stroke is a devastating condition that can result in lifelong disability or death, these consequences can be prevented [2]. According to the 2019 Global Burden of Disease (GBD) study, more than 90% of the stroke burden is caused by potentially modifiable risk factors. Controlling metabolic and behavioral risk factors could reduce more than 75% of this burden. Hypertension has been identified as the single most important modifiable risk factor for stroke [3–8], with more than half of all strokes worldwide attributed to hypertension [9, 10].

Primary prevention of stroke requires regular awareness campaigns, governmental education on risk factors, and individual control of blood pressure [11, 12]. Despite this, there is poor adherence to anti-hypertension treatment among hypertensive patients, even though they are aware that the disease is a serious risk factor for stroke. This is a significant concern as it suggests a wide gap between knowledge and practice of stroke prevention in this patient group [13]. According to a nationwide survey to assess the prevalence of hypertension in China, 23.2% (244.5 million) of the Chinese adult population had hypertension: despite a high prevalence of hypertension, awareness, treatment, and control of hypertension remain low [14]. Barriers still exist between patients and prevention strategies. Individuals’ perceptions of the disease are crucial in influencing actions toward prevention and adherence to treatment [15]. Studies have shown that the fear of being stigmatized can act as a barrier to the disclosure of a diagnosis [16–18].

Stigma has been described, based on the work of Goffman, as a quality that “significantly discredits” an individual in the eyes of others [19]. It is an attribute that reduces a person “from a whole and usual person to a tainted, discounted one” [*SIC*] [19]. Health-related stigma involves the stigmatization of a disease, which can be directed at an individual or a group of people with a specific condition, and to the illness more generally [20]. It is “characterized by exclusion, rejection, blame or devaluation that results from experience, perception or reasonable anticipation of an adverse social judgment about a person or group” [*SIC*] [21]. This phenomenon is not limited to patients with certain stigmatized diseases, but also affects the general public [19]. Stigma can be conceptualized as both external (public stigma) and personal (felt stigma). Public stigma, as described by Corrigan and Watson [22], encompasses the stigmatizing attitudes or reactions that the general population holds toward people with stigmatizing attributes, such as HIV infection or drug use.

Stigma can lead to negative consequences. Discrimination not only fosters fear and ignorance but also hampers the expansion of AIDS prevention and treatment services. Nevertheless, the success of AIDS prevention and treatment must be based on prevention and treatment services [23]. Meanwhile, the accessibility of cancer detection and prevention procedures (e.g., screening and HPV vaccination) requires people to consider the possibility of a cancer diagnosis. The fear of stigmatization has been identified as a potential barrier to self-examination, screening, and delayed presentation of cancer symptoms [24]. Moreover, research on Alzheimer’s disease has revealed that cultural stigma and misconceptions about the disease may contribute significantly to delays in diagnosis and treatment [25].

Stroke is a stigmatized disease, and this can be explained through a multidimensional concept of stigma. Jones et al. (1984) identified six components of health-related stigma, which apply to different degrees depending on the illness of interest [24]. The first component, “course,” refers to changes in the illness over time, with conditions that become progressively disabling, chronic and incurable becoming more stigmatized. This aspect is particularly relevant for stroke, as it is a major cause of long-term disability worldwide [26, 27]. The second component, “origin,” relates to when and how the illness is believed to have occurred. A particularly relevant aspect of this is the attribution of perceived responsibility. When a person is believed to have caused their illness, the stigma associated with it is greater [24, 28, 29]. This concept gains significance for stroke as lifestyle determinants are more widely recognized. The remaining four components are “peril” “concealability,” “disruptiveness,” and “aesthetics” (described as a primitive response by the perceiver to a non-concealable mark that makes the person less “pleasing on the eye”). These aspects are relevant to stroke, approximately 45% of stroke patients experience lasting disability [30]. In the community, stroke survivors often live with different degrees of physical, psychosocial, and cognitive challenges such as hemiplegia, aphasia, depression, or low self-esteem [31]. These six components help to highlight the aspects of the disease that may contribute to its stigmatization. Meanwhile, a high standard of stigma is characterized by personal guilt associated with acquiring the disease, the perception of it as incurable, a lack of knowledge about the disease, and symptoms that cannot be hidden [32]. Stroke possesses the majority of these characteristics.

Previous public stigma studies have found that stigma can prevent people from seeking or fully participating in health services [33, 34], and it can be a significant barrier to establishing widespread stroke health education. However, perceptions of stroke within the most important target for education, the hypertensive group, remain unknown. We aim to uncover and understand the perceptions of stroke among individuals with hypertension to address the critical need for targeted approaches to stroke prevention.

## Methods

### Theoretical Framework

Attribution theory, proposed by B. Weiner in 1972 [35], ranks as one of the central psychological theories to describe motivated behavior in humans [36, 37]. According to attribution theory, different attributions to the cause of the disease will lead individuals to have different emotional responses to the patient. This emotional response, in turn, affects the individual’s attitude and behavior toward the patient. In the attribution process, controllability is the most important factor. The individual’s perception of controllability determines the judgment of the patient’s responsibility for the disease, which is the main reason for the stigma and related behaviors. Individuals tend to attribute less responsibility, infer negative emotions, and express more sympathy for patients with uncontrollable causes than for those with controllable causes. Attribution theory emphasizes that health-related stigma is an unconscious emotional response, revealing the mechanism of stigma formation and how it is associated with specific groups. This perspective helps us better understand stigma on a personal level. It is also one of several social cognitive approaches to stigma that frame the phenomenon in terms of knowledge structures. This theory is especially appealing for understanding and changing stigma, as path models developed from it have mapped the relationship between signaling events, mediating knowledge structures (attributions), emotional reactions, and behavioral responses [38].

Attribution theory is fundamentally a model of human motivation and emotion based on the assumption that individuals search for a causal understanding of everyday events [37]. In the current study, attribution theory was used to design the outline and thematic analysis.

**FIGURE 1:**
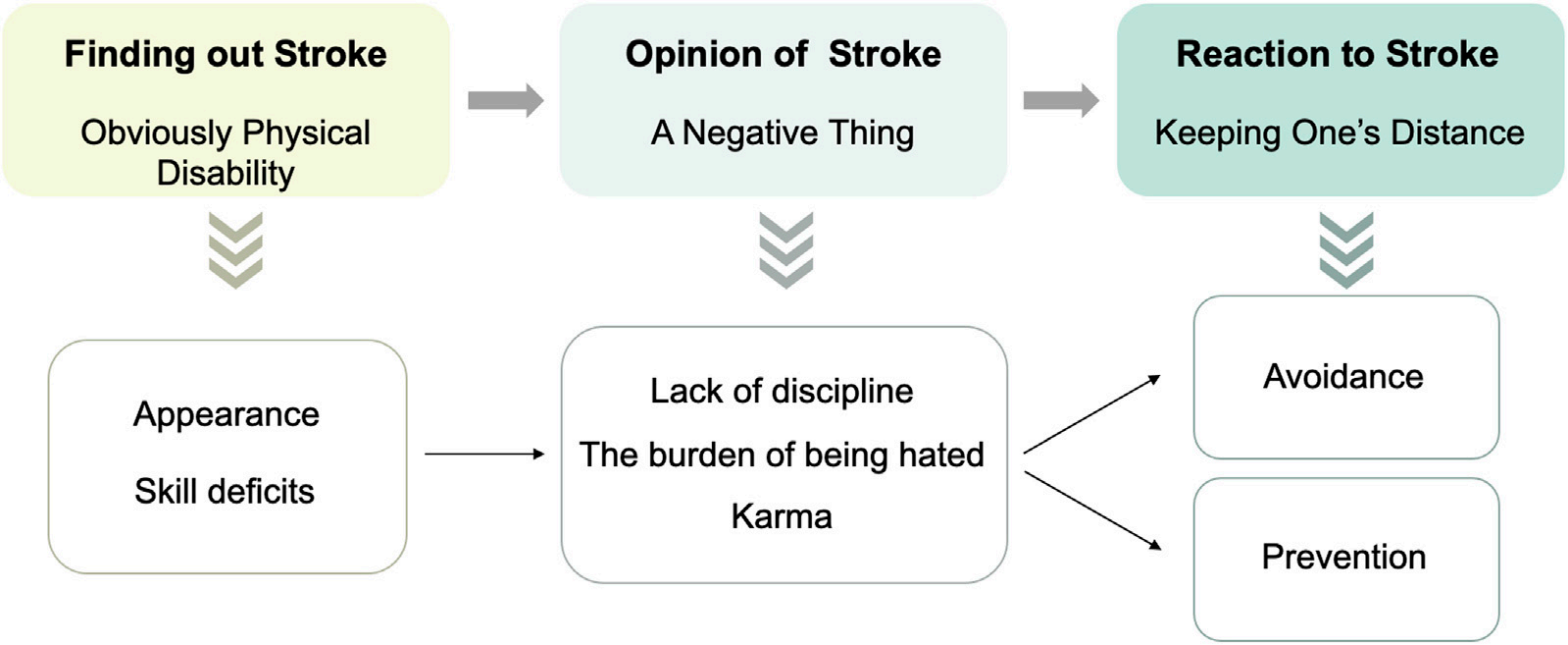
The relationship between signals (obvious physical disability), stereotypes (a negative thing), and behaviors (keeping one’s distance) (China, 2020).

As can be seen in Figure 1, the model generated from attribution research parallels the progression from an apparent physical disability—a negative thing—to a desire to keep one’s distance.

### Design and Participants

The methodological approach was qualitative. The study took place in urban China, and the COREQ-Consolidated criteria for reporting qualitative research [39] guidelines were used for reporting. Home-based participants with hypertension (N = 16) were recruited by purposive sampling to ensure sufficient gender variation and to prevent participants from being influenced by other stigmatized diseases. Inclusion criteria for the study were: diagnosed with hypertension by a clinician; aged >40 years; ability to understand language and express themselves clearly. The exclusion criteria were: suffering from other chronic diseases; cancer; infectious diseases; mental disorders (suicidal tendencies, depression, etc.); and having had a stroke.

### Data Collection

Data were collected using semi-structured interviews conducted by the first author (MW) prior to discharge. Pilot interviews were conducted to identify any unclear questions and to select key questions. The interviews were digitally recorded and transcribed verbatim. After analyzing 5 non-hypertensive college students and 3 hypertensive subjects—not included in the main study—three main questions were selected for the final study. The questions were:

(a) “What made you realize that these people were stroke patients?”
(b) “What caused them to have a stroke?”
(c) “How did it feel when you saw a stroke patient?”

Follow-up questions were used to elaborate on the answers when relevant. The 16 interviews lasted between 10 and 52 min (mean = 21.17 min).

All participants were informed verbally and in writing about the purpose of the study and the obligations of the researchers the day before the interviews. Due to COVID-19, video interviews were conducted from June to August 2020. The main researcher interviewed participants via WeChat, a software that facilitates communication between people.

The first author’s knowledge of stroke was based on a decade of life experience living with a stroke patient and 6 years of medical school. Additionally, the author participated in several community investigations on stroke and hypertension, and managed hypertension health in the community. Meanwhile, the research team that carried out this study has focused on the primary prevention of stroke for years.

### Data Analysis

The first author took notes from the interview, transcribed the data verbatim into a manuscript, and analyzed them thematically [40]. Following this, group discussions and brainstorming sessions were conducted. The interview manuscripts were then returned to the participants for comments and corrections. The data were imported into NVivo 12 for management and analysis. The researcher repeatedly read the interview manuscripts to gain a sense of familiarity and wrote down common identifying metaphors, statements, and ideas that were salient in revealing the participants' experiences and the meaning of facing stroke patients [41] (for example, how to identify stroke patients and the mental activities when meeting them). A total of 26 categories were identified. The codes related to the research question and appearing repeatedly in the interview manuscripts were summarized into subtopics and potential themes. Group discussions were conducted to demonstrate the consistency between the themes and the coding. A thematic map was then created, illustrating the interrelated subthemes and three major themes.

### Ethical Considerations

This study was approved by Xi’an Jiaotong university. Before the interview, participants were informed of their right to confidentiality and their right to withdraw from the study at any time. Their statements and personal identities were protected and anonymized during the publication process.

## Results

The characteristics of the participants are shown in Table 1. Analysis of the interviews revealed that the perceptions and behaviors of the hypertensive group toward stroke patients were complex and varied. During the interview, participants highlighted their past events, current health behaviors, and their feelings about stroke patients. Three themes and eight sub-themes were generalized in the analysis. The three themes were: Obvious physical disabilities; The negative image of stroke; *and* Keeping a distance from stroke. These are described in more detail below, exemplified through interview quotations (Table 2).

**TABLE 1:** Selected socio-demographics of the hypertensive population (China, 2020).

	(N = 16)
Sex	
Male subjects	8
Age, years	
40–50	5
51–60	5
61–70	2
71–80	4
Education level	
Primary school	5
Middle school	5
Junior college	3
College	3
Employed	
Yes	9

**TABLE 2:** Overview of categories, sub-themes, and themes from the content analysis (China, 2020).

categories	Sub-themes	Theme
Crooked mouth	Appearance	Finding out about stroke— obvious physical disability
Tilted head		
Gait dysfunctions		
Limb hemiplegia		
Aphasia	Skill deficits	
Slurred speech		
Dysphagia		
Mobility aids		
Incontinence		
Lifestyle	Lack of discipline	Opinion of stroke as a negative thing
Recurrence		
Neglectful of hypertension		
Witnessed death due to stroke		
Alcohol-related stroke incident and subsequent legal claims	The burden of being hated	
Blamed by family involvement		
Morality	Karma	
Lifestyle		
Reflection of potential risk	Examples of vigilance	
Concerns arising from the reflection on stroke incidence		
Social contact (positive way)	Avoidance	Reaction to stroke— keeping one’s distance
Avoidance of stroke information		
Feelings of despondency		
Social contact (negative way)	Prevention	
Heighten health vigilance		
Readiness for health assessments		

### The Conspicuous Physical Disabilities

The interviewees initially identified stroke patients by their physical disabilities, the first theme of the analysis. The two sub-themes that fell under this theme were *Appearance*, and *Loss of function.*

#### Appearance

Obvious physical disabilities are crucial for the public to distinguish stroke patients. Participants considered stroke symptoms such as an awkward gait, unbalanced limbs, and even a crooked mouth or tilted head. According to the interviewees, a typical stroke patient might resemble a stumbing child whose legs are consistently moving in irregular circles or kicking in the air. Their arms seem to be constantly holding some invisible object. Once such strange behaviors are observed, respondents can easily confirm who has suffered from a stroke.

“*Some of them have crooked mouth, or head tilted.*” [*SIC*] (*Woman, 80 years old*).

This interviewee has never lived with a stroke patient, she can only tell whether people have suffered from a stroke or not by their appearance.

#### Loss of function

We asked the respondents about the difficulties in the daily lives of stroke patients, and they mainly mentioned the loss of certain functions in daily life and social activities.

In daily life, stroke patients may need different levels of care, such as:

“*Food needs to be smashed up with a juicer and fed to him bit by bit.*” (*Woman, 73 years old*).

Interviewees mentioned that stroke patients can easily choke on food or water, and some of them can no longer walk. As a consequence, they are often confined to wheelchairs, pushed around by their family members, some of them being incontinent.

Socially, they have various impairments. For example, verbal communication, the most common means of interaction in any social activity, could become an impenetrable barrier if a patient is suffering from aphasia or slurred speech. Expressing themselves or making themselves understood would be an unimaginably difficult task for these patients.

“*I*”*m so worried for him, he wants to express something, but he cannot say a word*. [*SIC*] (*woman, 73 years old*).

*He spoke for a long time, but he did not know how to express it, and his language was broken.* [*SIC*] (*man, 68 years old*).

Language difficulties can make interviewees give up trying to communicate with stroke patients.

### The Negative Image of Stroke

The second theme is the negative image of stroke. Largely due to personal experience and understanding of the disease, people regard stroke as a serious disease with significant negative effects, and its elusive cause has always been a hot topic. The three sub-themes that fell under this theme include the *lack of discipline*; *the feeling of being an undesired burden,* and *karma*.

#### Lack of Discipline

Participants attributed the cause of stroke to an unhealthy lifestyle. With the promotion of large-scale health education, an increasing number of people are realizing the importance of a healthy lifestyle. The majority of the interviewees are aware that a healthy lifestyle can help prevent stroke, and some respondents believe that stroke can be prevented through personal habits and an awareness of the harm caused by bad habits.

“*Life-style is very important, for example, eating too much, especially too much greasy food, is not good for our body. As an old saying goes, illness finds its way in from the mouth, so you must manage your diet and exercise more.*” *[SIC]* (*woman, 73 years old*).

“*Compared with diet, I think smoking, drinking, and staying up late are the most important cause of a stroke, especially staying up late.*” [*SIC*] (*man, 53 years old*).

“*I used to drink alcohol during social gatherings, and I have a long smoking history. I did not quit smoking until 2006. I am now aware of the harm caused by smoking and drinking.*” [*SIC*] (*man, 68 years old*).

Some of the interviewees are also aware of the recurrence of stroke attacks.

“*This is the sixth time he has had an attack, the sixth time!*” [*SIC*] (*woman, 73 years old*).

A sense of blame could be discerned from their tone. The symptoms of the first stroke attack were usually mild and failed to attract enough attention in the minds of the patients. They would dismiss it after recovery and continue their unhealthy habits until the disease recurred and hit them again.

There are also friends of our interviewees who had strokes because they ignored their hypertension. Moreover, some of the interviewees have witnessed many severe stroke events around them, including some fatal strokes.

“*My third brother had a cerebral hemorrhage, it’s a brainstem hemorrhage, and it was very fast. Eventually, he could not shake it. After dinner, he told his wife that he had a headache, then he fell down and died when he was sent to the hospital.*” [*SIC*] (*woman, 46 years old*).

These life-threatening events had a significant impact on hypertensive interviewees. They may have thought that stroke was just a chronic condition before suddenly realizing that it was a deadly threat, and a wave of fear would follow.

#### The feeling of being an undesired burden

Participants directly or indirectly stated that stroke patients are a burden and are loathed. Personal experiences with stroke patients strongly influence hypertensive patients’ perceptions of stroke. When the interviewees discussed stroke patients, their facial expressions subtly shifted, displaying nuances such as furrowed brows or smiles. Some participants have experienced or heard about some unpleasant events in their lives with stroke patients, which will affect these people’s views on stroke and their attitudes toward stroke patients, such as experiences involving sudden stroke attacks and subsequent financial disputes, and this may have a direct or indirect impact on the group.

“*Once there was a stroke attack when I was eating with him, we had a good relationship. Then his family blamed me for his stroke attack and put forward many conditions. I was under a ton of pressure, in the end, I had to pay him a lot of money.”* [*SIC*] (*man, 68 years old*).

Furthermore, the burden of stroke on family members can be daunting. The responsibility of caring for stroke patients falls mainly on family members. Older people and even middle-aged people with children are sometimes worried that they will become a burden to their families. Knowing that they may also have a stroke problem could greatly increase this worry, so the majority of participants will blame stroke patients for being a heavy burden on their families.

#### Karma

Many Chinese believe in the concept of reaping what one sows. This old saying could be epitomized in a Buddhist term called Karma. It can be understood in two ways: one is moral, and the other is healthy behavior. First, we will be punished for doing bad things, which encourages us to do good things.

“*A man of honor would take responsibilities when the situation is serious, be sincere to every beings, have kind thoughts, refrain from thinking about insidious deeds, or be covetous for small gains, and eventually, good will would be rewarded.*” “*Nobody else but you should be the one who will get sickness if you only think about bad things all day and always do bad things.*” [*SIC*] (*man, 68 years old*).

On the other hand, the participants also thought that the cause of stroke was tied to unhealthy behavior. In general, from the point of view of morality and healthy behavior, people thought that the stroke patients themselves were responsible for their disease.

### Keeping a Distance From Stroke

The third theme is keeping the stroke at arm’s length. Upon discovering a person with a stroke disease, people will make judgments based on their own feelings about the stroke and eventually display certain behaviors. The two sub-themes that fall under this theme are *Avoidance* and *Prevention*.

#### Avoidance

People with high blood pressure want to keep their distance from stroke, which is easy to understand, but sometimes escaping is not the right solution to the disease. It can even lead to depression and worry.

“*If I got a stroke, I would rather to die.*” [*SIC*] (*man, 70 years old*).

With this feeling, the respondents always want to avoid stroke patients. They are afraid of the disease.

“*Over time, the relationship will inevitably be alienated, though not deliberately. I cannot stay with the patient every day. Try not to get sick, and if you do get sick, you will become a miserable shadow of your former self.*” [*SIC*] (*man, 68 years old*).

#### Prevention

In contrast to avoidance, some of the interviewees’ ways to stay away from stroke are positive and optimistic.

“*I would like to chat with stroke patients to learn about their lifestyle and bad habits, which maybe the cause their disease. Thus I could learn how to prevent stroke.*” [*SIC*] (*woman, 80 years old*).

Many participants will pay more attention to their health after seeing stroke patients. They will look at their behavior in the hope that they can be healthier, and some interviewees are willing to participate in community stroke screening programs. As a high-risk group, our participants are clearly aware of their association with stroke, so when they see stroke patients, they will have more complicated feelings that make them vigilant. They think about themselves when they see a stroke patient.

“*Seeing them is just like looking in a mirror.*” (*woman, 73 years old*).

## Discussion

The obvious physical disabilities caused by a stroke are easily recognized by hypertensive participants, and they perceive a stroke as a negative thing. Recognizing the link between hypertension and stroke, participants express a desire to maintain a distance from the stroke. However, their strategies for achieving this distance are complex, diverse, and at times contradictory—ranging from avoidance to preventive measures.

More than 85% of people survive a stroke, and of these, more than 80% are discharged to the community [42]. Their symptoms are visible to the public and are likely to lead to a situation in which the individual is excluded from full social acceptance, as defined by Goffman in 1963 [19], which is an important reason why hypertensive patients can recognize that stroke patients are “different.” Studies [43, 44] have shown that many people understand that hypertension is the main risk factor for stroke, and people with hypertension themselves are generally aware that their disease is related to stroke. The perceived origin of a disease is an important component of stigma [24]. According to Jones et al. (1984), the concept of origin encompasses perceptions about the responsibility of the individual for contracting the stigmatizing condition. “Attributions about why a negative event occurred, especially the stigmatized person’s perceived control over the cause, have great influence over how others will think of and behave toward that person” [45] (p. 513). In the current study, we found that participants believed that stroke patients should take responsibility for their disease and viewed stroke as a lack of discipline. Meanwhile, culture contributes significantly to shaping the health beliefs and attitudes of individuals. In this study, participants thought they should not treat the disease as a moral punishment, but they actually thought it was true deep in their minds. This finding is supported by other studies. People perceive Alzheimer’s disease as a punishment from God [46]. Some subjects diagnosed with stroke perceived stigma against these conditions because of their association with curses and evil deeds [47]. Some people believe that the reason a person gets cancer is perceived as a result of witchcraft and karma [48, 49]. In addition, in China’s traditional culture, family members always take care of the diseased [50], with the illness seen often as a burden to the entire family, not just an individual. All of these contribute to the feeling of fear in the hypertension group.

China has been conducting stroke health education programs for many years, such as the “Stroke 120” campaign [2], and many other educational interventions launched in recent years [51–55]. All of these studies have made great progress, but the current study, as one of its limitations, found that a group of hypertensive individuals may avoid addressing stroke altogether. They simply do not want to hear about stroke, and they are afraid of it. This explains why the existing intervention methods have blind spots.

Perhaps the answer is in other studies of health-related stigma. A meta-analysis found that both contact with the patients and education seem to significantly improve attitudes and behavioral intentions toward people with mental illness, with face-to-face contact with the person, rather than a videotaped story, having the greatest effect among adults [56]. The role of media is vital; appraisal theory argues that emotions arise from automatic cognitive evaluations of a situation, with each emotion distinguished from another by its specific pattern of cognitive appraisals [57, 58]. Appraisal theory predicts that the action tendency associated with sadness is introspection [43]. One study found that empathy could reduce stigma toward people with AIDS, homeless people, and murder convicts [59]. A study on cancer found that compassion was indirectly related to individual behavioral intentions [60]. Another study indicated that the most important sources of information were the internet and television [46].

The participants in this study are very worried about becoming a burden to their families, but perhaps families can be motivated. The impact of stigma is magnified by the fact that stigma is attached not only to the individual with mental illness but also to the individual’s family [61]. Individuals always manage their personal affairs with the help of support networks formed by family ties. Family emotional support plays an important role in promoting subjective support and requires further research [50]. Meanwhile, peer support is crucial. Studies have indicated that peer support may contribute to the prognosis of stroke by facilitating connections between stroke survivors and like-minded individuals in the community [62, 63]. Peer support also plays a positive role in hypertension control [64]. Research suggests that peer support may enhance the scope of comprehensive care, contributing to the sustainable development of healthcare systems [65].

Therefore, educational programs should be planned with the spirit that the approach needs to be changed. The hypertension group in this study wants more ways to prevent the disease, not just manage the stroke. Education can start this way; behavioral/lifestyle modifications have been shown to contribute to up an 80% reduction in the risk of stroke [66]. To bridge effective knowledge to people with high blood pressure, we need to increase our use of the media, increase sympathy, and encourage the interaction of patients with hypertension and stroke in the community. At the same time, we should encourage hypertensive patients who are actively acquiring knowledge about stroke prevention to share it with their friends so that education can be more widely disseminated and percolate into the population through the power of peers. Encourage a shift in approach for those passively avoiding the mention of the disease. Utilizeindirect communication about stroke and effectively leverage familial channels for educational purposes.

We acknowledge that in some instances in our study, despite our best efforts to clarify and explain, some respondents understanding of the stroke conditions under study may have been unclear. At the same time, he also thought that after talking to him about stroke, he would constantly think about the disease, and he might get a stroke. Hence, the participants selected for this study were individuals who demonstrated a willingness to discuss stroke, indicating a moderate level of openness and receptiveness to the topic. Despite these limitations, we believe that the study provides an important perception of stroke.

### Conclusion

People with hypertension exhibit contradictory behaviors that they believe will help them stay away from stroke, as there is a certain stigma associated with stroke. In the future, we should focus on changing the way we educate hypertensive groups through public health and health educational programs, for instance by improving media promotion, and encouraging interaction with patients with hypertension and stroke in the community.
